# Hydrogenotrophs-Based Biological Biogas Upgrading Technologies

**DOI:** 10.3389/fbioe.2022.833482

**Published:** 2022-04-25

**Authors:** Tatsiana Antukh, Ingyu Lee, Sunghee Joo, Hyunook Kim

**Affiliations:** Water-Energy Nexus Laboratory, Department of Environmental Engineering, University of Seoul, Seoul, South Korea

**Keywords:** biogas upgrading, biological hydrogen methanation, hydrogenotrophic methanogens, renewable energy, biogas acceptance

## Abstract

Biogas produced from anaerobic digestion consists of 55–65% methane and 35–45% carbon dioxide, with an additional 1–2% of other impurities. To utilize biogas as renewable energy, a process called biogas upgrading is required. Biogas upgrading is the separation of methane from carbon dioxide and other impurities, and is performed to increase CH_4_ content to more than 95%, allowing heat to be secured at the natural gas level. The profitability of existing biogas technologies strongly depends on operation and maintenance costs. Conventional biogas upgrading technologies have many issues, such as unstable high-purity methane generation and high energy consumption. However, hydrogenotrophs-based biological biogas upgrading offers an advantage of converting CO_2_ in biogas directly into CH_4_ without additional processes. Thus, biological upgrading through applying hydrogenotrophic methanogens for the biological conversion of CO_2_ and H_2_ to CH_4_ receives growing attention due to its simplicity and high technological potential. This review analyzes the recent advance of hydrogenotrophs-based biomethanation processes, addressing their potential impact on public acceptance of biogas plants for the promotion of biogas production.

## 1 Introduction

Over the last 2 decades, the bioenergy sector has received increasing attention, especially in the usage and production of biogas. The number of facilities producing biogas *via* anaerobic digestion (AD) processes has increased steadily. Germany is a leader in terms of biogas plants. Currently, about 9,000 farm-scale digesters are operating in the country (Vasco-Correa et al., 2018). In the US (U.S. EPA, 2017 & 2018), there are a total of 209 anaerobic digesters fed with food-waste and 1,250 anaerobic digesters fed with wastewater sludge. In 2017, Australia had 242 biogas plants, half of which were on landfill sites (Carlu et al., 2019). In Denmark, 150 biogas-producing plants were operating in 2015 (EBA, 2015). According to Kalyuzhnyi (2008), there were around 100 anaerobic digesters in Russia in 2008. From 2012 to 2020, the number of biogas plants in the Republic of Korea increased from 49 to 110 (Kim et al., 2012; Korean Ministry of Environment, 2020).

In China, the amount of wastes treated by the AD process increased from 21,600 tons per day in 2015 to 36,400 tons per day in 2020 (Khalid et al., 2020). Nonetheless, actual biogas production is only about 6% of the potential for China (35 Mtoe vs. 570 Mtoe), according to the International Energy Agency (IEA) (2018). In India, approximately five million small-size family biogas plants have been installed, but only 56 biogas-powered plants are operating (Mittal et al., 2018). It appears that the biogas production and usage has a great potential for development and application. Although the number of biogas plants has increased, the produced biogas has been limitedly utilized to produce electricity or heat for homes or towns in the vicinity. It is mainly because the amount of biogas produced by a plant is not large enough to supply to industrial plants and the biogas is not pure enough to directly supply to a gas grid or automobiles without further purification. Therefore, biogas upgrading to biomethane, i.e., biogas mainly consisting of methane, has recently received particular attention from biogas producers.

The composition of biogas produced during AD is around 55–65% methane and 35–45% carbon dioxide, similar to landfill gas (Oslaj et al., 2010; Nasir et al., 2012; Ounnar et al., 2012). To meet the requirements to be used as biofuel (e.g., for gas-powered vehicles), biogas must be purified to increase the methane gas content (ISO 13686:1998(en) Natural gas—Quality designation, 1998). Thus, biomethane is supplied to natural gas facilities and used directly as a raw material for energy production and the chemical industry.

Biogas upgrading aims to remove or separate the carbon dioxide and other impurities from the biogas to achieve a methane content of up to 95%, thereby securing heat at the natural gas level and further utilization as a fuel (Sun et al., 2015). One of the purposes of biogas upgrading is to make biogas a stable energy source and an alternative to fossil fuels (Lecker et al., 2017). Additionally, the upgraded biogas can be injected directly into existing gas pipelines with no extra processes required. However, issues such as unstable production of high-purity methane gas, high operation costs, large facility size, and high energy consumption during the upgrading process are still a challenge in biogas upgrading that must be resolved (Ahern et al., 2015; Adnan et al., 2019).

The application of conventional biogas upgrading includes many scrubber processes that utilize water or amine as an absorbent or use pressure swing adsorption and membrane separation (Angelidaki et al., 2018; Struk et al., 2020; Nguyen et al., 2021). Although the membrane-based upgrading process has high energy efficiency and is easy to operate and maintain, additional capital investment is required for the installation of compressors, membrane modules, heat exchangers, and off-gas treatment devices (Angelidaki et al., 2018; Nguyen et al., 2021). Furthermore, a large amount of energy is required to achieve a high level of purity of methane, which is the issue to maintain operating costs in an acceptable range (Angelidaki et al., 2018; Nguyen et al., 2021).

In addition, physical condensation and chemical adsorption or absorption methods are applied mostly to remove moisture, H_2_S, ammonia, and other trace elements. To remove CO_2_ from biogas, additional technologies (e.g., chemical absorption, water scrubbing, cryogenic separation, membrane separation, or pressure separation) are necessary (Ryckebosch et al., 2011; Muñoz et al., 2015; Awe et al., 2017). The use of physical and chemical methods has many disadvantages, including, but not limited to, high energy consumption, difficult operation, CH_4_ loss during purification, and a high cost of investment and operation (Awe et al., 2017). Compared to those technologies, biological upgrading technologies overcome these problems (Khan et al., 2021).

With specific microorganisms known as hydrogenotrophic methanogens, conversion of CO_2_ into CH_4_ is possible, allowing an increase in CH_4_ content of up to 95% and meeting natural gas standards (ISO 13686:1998(en) Natural gas—Quality designation, 1998). Recent research has demonstrated that hydrogenotrophs-based biological methanation (HBM) could be a promising technology for biogas upgrading (Singhal et al., 2017; Adnan et al., 2019). In fact, HBM has been demonstrated to be the most effective way of converting excess electricity into natural gas to avoid energy losses (Lecker et al., 2017). Based on the findings, Luo and Angelidaki (2012) proposed excessive hydrogen utilization *via* biological biogas upgrading. The study by Adnan et al. (2019) has reviewed different biogas upgrading techniques and found HBM as a good potential for sustainability, cost-effectiveness, and environmental impact, although the development of biological upgrading is still in its early stage.

However, due to its novelty, there are just a few case studies concerning biological methane upgrading in large-scale systems (IEA Bioenergy, 2018; Jensen et al., 2018; Lebranchu et al., 2019). Additionally, since HBM is a developing technology, there are only a few studies focusing on review of the biological upgrading processes (Lecker et al., 2017; Zabranska and Pokorna, 2018; Voelklein et al., 2019; Fu et al., 2020).

This review examines biogas upgrading systems utilizing hydrogenotrophic methanogens. For the first time, this review explores the microbial pathways of hydrogenotrophic methanogens involved in the biogas upgrading to biomethane. The pros and cons of the different biogas-upgrading system configurations are analyzed, along with methods to improve H_2_ transfer and the operational conditions. Perspectives for the improvement of public acceptance of biogas production are discussed, and directions for future research are suggested.

## 2 Biogas Upgrading *via* Hydrogenotrophic Methanogens

Biogas upgrading, as a way of increasing methane content in biogas, is performed by 1) removing CO_2_ and other trace gas components (water vapor, siloxane, hydrogen sulfide, ammonia, oxygen, nitrogen) from biogas through the additional physical/chemical processes attached to the AD process (Muñoz et al., 2015; Awe et al., 2017; Adnan et al., 2019); 2) by converting CO_2_ from biogas to methane (Lecker et al., 2017; Adnan et al., 2019; Fu et al., 2020). Hydrogenotrophs-based biological biogas upgrading technologies are performed by converting CO_2_ in biogas to methane and utilizing specific microorganisms called hydrogenotrophic methanogens [e.g., *Methanosarcina barkeri*, *Methanogenium frittonii*, *Methanomicrobium mobile* (Dworkin et al., 2006); *Methanothermus fervidus; Methanobacterium bryantii; Methanothermobacter thermautotrophicus* (McGenity et al., 2010)]. Due to the technical simplicity, the technologies have been widely utilized. Biological upgrading is attractive in terms of 1) biogas purification; 2) an environment-friendly technology by capturing CO_2_ and converting it to CH_4,_ a source for electricity production; 3) simplicity; and 4) easy operation (Fu et al., 2020). Nonetheless, there are a number of issues to be resolved for wide applications of the technology. In this section, technical aspects of the hydrogenotrophs-based biological biogas upgrading are reviewed and discussed.

### 2.1 Microbial Pathways for Biogas Upgrading and HBM System Configurations

There are two main methanogens groups in the anaerobic digester—acetoclastic methanogens (AMs) (using acetic acid as a substrate), and hydrogenotrophic methanogens (HMs) (using hydrogen and carbon dioxide as substrates). Both types convert the substrates into methane (Jones et al., 1987; Tian et al., 2019; Conrad, 2020). Biological biogas upgrading primarily applies to microbial communities of HMs. Because additional processes are not necessarily required, relatively little energy is consumed compared to other technologies (Angelidaki et al., 2018). This type of methanogens can also influence the operation efficacy of an AD reactor.

**FIGURE 1:**
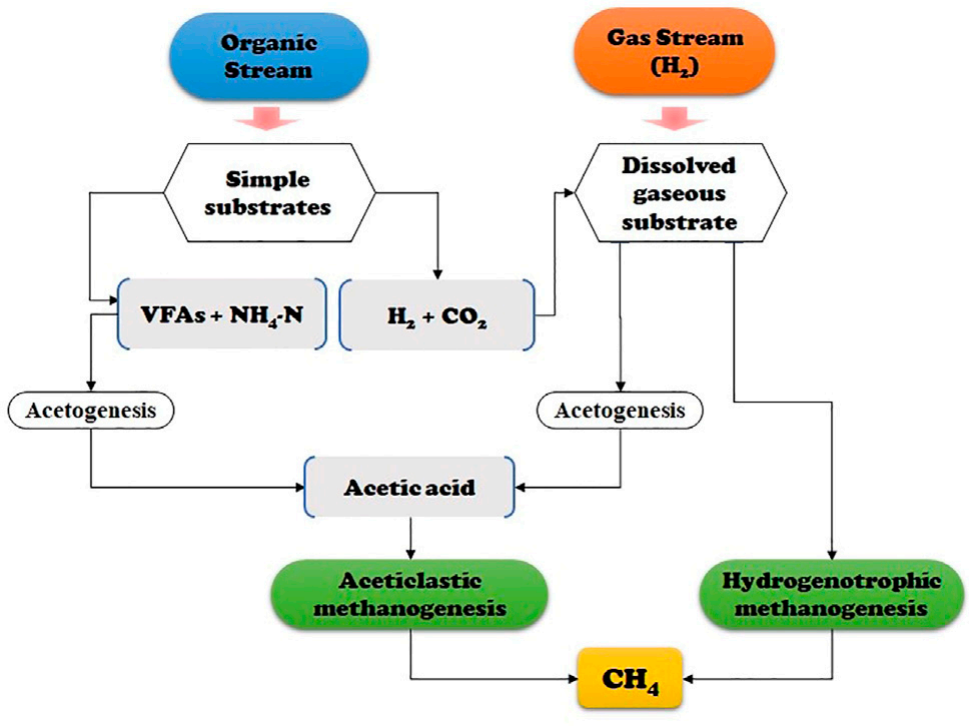
Schematic diagram of the acetate-oxidizing bacteria assisted metabolic pathway *via* H_2_-substrate and hydrogenotrophic methanogenesis.

HBM upgrading provides additional methane production through combining H_2_ and CO_2_ *via* the metabolic pathway under either mesophilic or thermophilic conditions (Ahern et al., 2015; Guneratnam et al., 2017; Zabranska and Pokorna, 2018). The principal schematic of biological pathways of the process is presented in Figure 1. When using microorganisms, usually both AMs and HMs are applied to produce high-purity biomethane. The primary reaction pathways of HBM include 1) conversion of CO_2_ and H_2_ to CH_4_ by HMs (Eqs 1, 2) methanation of acetic acid by AM activity (Eqs 2, 3) generation of acetic acid through a homoacetogenesis reaction due to the high substrate activity (the Wood-Ljungdahl pathway) (Eq. 3), as shown in the following equations (Thauer et al., 1977; Ragsdale and Pierce, 2008).

$${4\text{H}}_{2}{+\text{CO}}_{2}⇌{\text{CH}}_{4}{+2\text{H}}_{2}\text{O}\;\;\Delta {\text{G}}^{\text{o'}}{=\;-131\text{ kJ mol}}^{-1}\left(\text{T}=298\text{K;}\;\text{ P}=1\;\text{atm;}\;\text{pH}=7\right)$$ (1)

$${\text{CH}}_{3}{\text{COO}}^{-}{+\text{H}}^{+}⇌{\text{CO}}_{2}{+\text{CH}}_{4}\;\;\Delta {\text{G}}^{\text{o'}}{=-36\text{ kJ mol}}^{-1}\left(\text{T}=\text{298 K; P}=1\;\text{atm;}\text{ pH}=7\right)$$ (2)

$${4\text{H}}_{2}{+2\text{CO}}_{2}⇌{\text{CH}}_{3}{\text{COO}}^{-}{+\text{H}}^{+}{+2\text{H}}_{2}\text{O}\;\;\Delta {\text{G}}^{\text{o'}}{=-95\text{ kJ mol}}^{-1}\left(\text{T}=\text{298K; P}=1\;\text{atm;}\text{ pH}=7\right)$$ (3)

**FIGURE 2:**
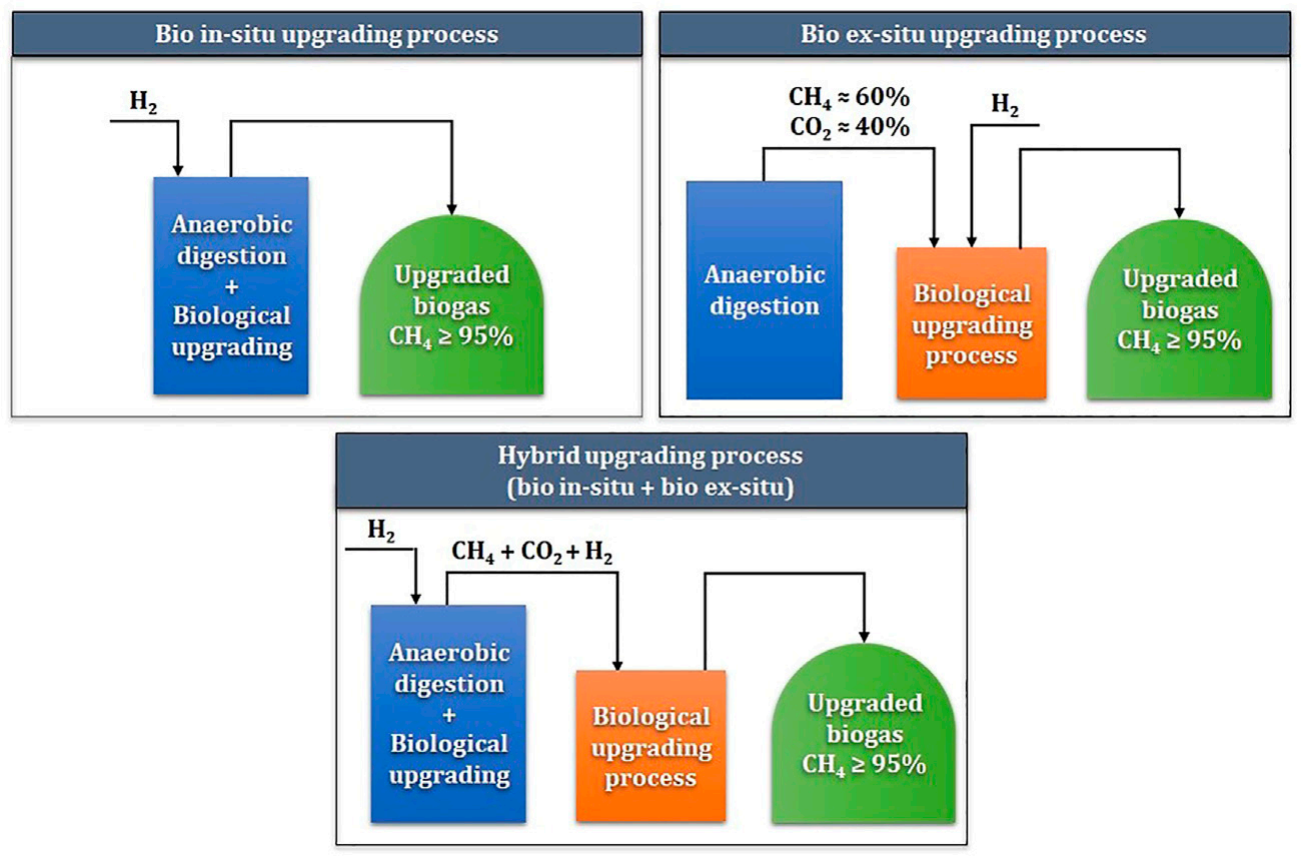
Conceptual diagram of *in-situ*, *ex-situ*, and hybrid biological hydrogen methanation processes.

Hydrogenotrophs-based biological methanation is classified into *in-situ*, *ex-situ*, or hybrid biogas upgrading, depending on the reactor configuration and the injection of H_2_ and CO_2_ (Lecker et al., 2017). Figure 2 demonstrates the *in-situ*, *ex-situ*, and hybrid processes of HBM. For *in-situ* biogas upgrading, H_2_ gas is injected directly into an AD reactor and converted to methane by HMs, along with CO_2_. Together, AMs convert volatile fatty acids (VFAs) into methane in the same reactor (Lecker et al., 2017; Angelidaki et al., 2018). On the other hand, in *ex-situ* biogas upgrading, H_2_, CO_2_, and CH_4_ gases are injected at a separate or stand-alone reactor with a single culture of HMs (Lecker et al., 2017; Angelidaki et al., 2018). Although the *ex-situ* process overcomes various biological and mechanical challenges, separate reactor construction is required, which might be undesirable for plants with limited space.

Hybrid systems combining both *in-situ* and *ex-situ* technologies can be achieved for HBM. These biological biogas upgrading technologies combine technological advantages and avoid the disadvantages of a purely *in-situ* or *ex-situ* process. The hybrid systems can be performed as a combination of biological processes only or as a combination along with physical-chemical processes (Khan et al., 2021; Nguyen et al., 2021). Whereas hybrid systems appear the most efficient for biomethane production, high investment and complexity make the technology less attractive compared to solely *in-situ* or *ex-situ* processes.

HBM systems have the potential to produce up to 95% CH_4_ content in continuous hydrogen injection systems (Corbellini et al., 2018; Voelklein et al., 2019), yet the reaction between cells and gases in the biomethanation process may not occur sufficiently due to the solubility difference between H_2_ and CO_2_; the solubility of H_2_ in water (1.6 mg L^−1^; Kaye and Laby, 1986) is extremely lower, compared to that of CO_2_ (1.7 g L^−1^). In addition, the accumulation of VFAs in the system may decrease pH and cause the reactor to be disturbed (Corbellini et al., 2018). Thus, H_2_ supply to the system needs to be adjusted optimally to maintain the metabolic pathways of biological upgrading. Advantages and disadvantages of HBM system configurations are analyzed in the following sections.

### 2.2 Pros and Cons of *In-Situ* Biogas Upgrading Technologies

In *in-situ* HBM upgrading process H_2_ is supplied directly into the AD reactor. Depending on operational conditions, reactor configurations, and the substrates supplied, the AD reactors vary widely, creating a great variety of upgrading technologies. Furthermore, additional system constructions are not required for the *in-situ* process and essential CO_2_ and H_2_ are generated during the AD process. Despite various benefits to *in-situ* upgrading, the main drawback is difficulties in supplying the additional H_2_ to maintain the proper H_2_/CO_2_ ratio and finding the best way to supply H_2_ to increase gas-liquid transfer. Other issues with *in-situ* biogas upgrading include 1) the low solubility of H_2_; 2) changes in the microbial pathway from the addition of H_2_, causing operational issues or even reactor failure if not properly controlled; 3) CO_2_ depletion and pH increase through excessive H_2_ input (Corbellini et al., 2019).

An HBM method of biological binding of H_2_ is presented in a study by Luo et al. (2012), specifically a process of converting CO_2_ into CH_4_ through the supply of H_2_ as an electron donor by HMs. A biological methanation study with an *in-situ* lab-scale reactor performed by Luo and Angelidaki (2012) demonstrated that the CH_4_ generation rate during the stabilization period of 10 days was 1.5–5.3 L L_reac_^−1^ d^−1^, rising to 6–24 L L_reac_^−1^ d^−1^ as the amount of H_2_ injection was increased. Nonetheless, the CH_4_ content was maintained at 90–95% all the time. A CH_4_/H_2_ yield of 0.23, which is slightly lower than the theoretical CH_4_/H_2_ yield of 0.25, was reported, suggesting that most of the supplied H_2_ was used to produce CH_4_. Since the operation system was thermophilic, the reactor was maintained at 55°C, with pH maintained at around 7.8, while mixing at 500–800 rpm was supplied to the reactor (Luo and Angelidaki, 2012). *Methanobacteriales* was found the dominant order in the microbial community, with 90% CH_4_ content. The study also demonstrated that the gas-liquid mass conversion of H_2_ would limit the performance of HBM, thereby obtaining the conversion rate of 130 ml H_2_ min^−1^, which corresponds to the H_2_ injection rate of up to 24 L L_reactor_^−1^ d^−1^.

#### 2.2.1 Advance of H_2_ Transfer

As aforementioned, the low solubility of H_2_ limits its availability for microbial reactions in biological upgrading. One of the ways to overcome this issue is to improve the gas-liquid transfer through changing reactor configurations such as increase of gas pressure and by the installation of proper H_2_ diffusion systems. Different studies investigated different diffusers to improve the H_2_ transfer. For example, Bassani et al. (2016) applied upgrading to the up-flow anaerobic sludge blanket (UASB) thermophilic AD reactor treating potato-starch wastewater. Results indicated that the CO_2_ content in the biogas decreased from 42 to 10%, and that the final biogas was upgraded from 58 to 82% CH_4_ content. This was achieved by distributing H_2_ through a metallic diffuser, followed by a ceramic sponge in a separate chamber, and by having a volume of 25% of the reactor, and by applying a mild gas recirculation. Lebranchu et al. (2019) carried out a study on the HBM process, in which they used a 100 L pilot-scale digester with a dense membrane for H_2_ injection. In their study, residual H_2_ from high H_2_ injection flow rates was found at the digester outlet, indicating a transport limit caused by dissolved CO_2_ rather than by H_2_ mass transfer. In addition, their study regarding the effects of agitation rate on gas-flow rates revealed that, although hydrogen transfer was improved by its injection into the membrane, it was still highly affected by the agitation rate.

Recently, membrane diffusers have been applied to supply H_2_ gas to an HBM process. Especially, a hollow fiber membrane (HFM) has been applied for AD processes for the purpose of dissolving more H_2_ in the mixed liquor and producing more biomethane (Luo and Angelidaki, 2013; Wang et al., 2013; Alfaro et al., 2019). The newly developed system consisting of simultaneous coke oven gas (COG) biomethanation and *in-situ* biogas upgrading, showed high final CH_4_ content levels, up to 98–99% (Wang et al., 2013). The direct injection of COG, consisting of 92% H_2_ and 8% CO, into the anaerobic reactor through an HFM appears to be a highly efficient way of treating sewage sludge at a controlled pH of 8, with no apparent negative effects. However, the addition of COG could influence the structures of both the bacteria and archaea communities in the liquid.

Several studies revealed that the biofilm formed on the membrane contributed to the biological conversion of H_2_ and CO_2_ to CH_4_ (Luo and Angelidaki, 2013; Wang et al., 2013; Alfaro et al., 2019). Notably, their studies found that the majority of H_2_ was still utilized by microorganisms in the liquid. A study by Luo and Angelidaki (2013) demonstrated an increase in the generation of CH_4_, up to 96% in the upgraded biogas, and another study showed an increase of CH_4_ production by 42% compared to conventional digestion (Alfaro et al., 2019). The effect of biofilm on CH_4_ production through the biological conversion of H_2_ and CO_2_ needs further evaluation in terms of mechanisms, operational conditions, and environmental factors.

Table 1 summarizes the efficiency of *in-situ* systems with different operating conditions. *In-situ* HBM systems are based on the type of substrate and diffuser, operational temperature, pH, and H_2_ supply rate. Among different systems, the final CH_4_ content in the biogas was highest from the use of an HFM diffuser, taking it to 98.8% CH_4_ content (Luo and Angelidaki, 2013; Wang et al., 2013), indicating that H_2_ transfer can be successfully improved by the installation of an HFM diffuser. However, given that the *in-situ* reactor is operated with a mixed culture of AMs and HMs, a negative impact on gas transfer to the HMs is predicted, caused by the presence of highly concentrated organic waste and their derivatives (e.g., VFAs).

**TABLE 1:** Comparison on the efficiency of *in-situ* methanation systems.

Operation conditions				Performance result					Comments	References
Reactor type	Substrate	Diffuser type	Diffuser pore size	pH	Temp., °C	CH_4_, %	CH_4_, L/L/d	CH_4_ yield, L/kg VS		
CSTR	Cattle manure, Whey	Ceramic	14–40 µM	7.9	55	75	0.89		The smaller pore size of the diffuser resulted in higher efficiency of H_2_ consumption and CO_2_ conversion	Luo and Angelidaki (2013)
CSTR	Cattle manure, Whey	Column	0.5–1 mm	7.7	55	53	0.76			
CSTR	Cattle Manure	Ceramic	—	8.1	55	63.5	0.37		More than 90% of added H_2_ was consumed. Partial pressure and mixing intensity were the most important factors in affecting H_2_ consumption	Luo et al. (2012)
CSTR	Cattle Manure	Not specified	—	7.8	35	89	0.1	168	Mesophilic conditions showed worse efficiency compared to thermophilic (% is relatively similar but volume is much lower)	Bassani et al. (2015)
CSTR	Cattle Manure	Not specified	—	7.9	55	85	0.36	359		
CSTR	Pig manure	Not specified	∼1.5 mm	7.6	35	70		210	The thermophilic system showed better performance. Further, it was found that continuous stirring did not have a negative effect on the thermophilic reactor, which is the opposite result to the mesophilic reactor	Zhu et al. (2019)
CSTR	Pig manure	Not specified	∼1.5 mm	7.8	55	78		245		
CSTR	Sewage sludge	HFM	0.4 µM	8.1	35	73	0.54		Mesophilic conditions still increased CH_4_ content in the biogas. In addition, with gas recirculation get better upgrading results were achieved	Alfaro et al. (2019)
CSTR	Sewage sludge	HFM		8	37	98.8	0.65	220	COG gas was injected together with H_2_, resulting in the highest CH_4_%, but the lowest yield as a volume	Wang et al. (2013)
Batch	Glucose	—	—	7.6	37	94.5	0.04			Wahid et al. (2019)
Batch	Grass	Fish stone	Not specified	8	55	32	1.82	460	Very low CH_4_%, but yield is higher than average	Voelklein et al. (2019)
Batch	Grass	Ceramic	Not specified	8.4	55	60	2.52	640	Grass is the best sub. for CH_4_ production by volume	
UASB	Potato-starch	Rashig rings + Alumina ceramic sponge	Not specified	7.8	55	66	1.37		Gas recirculation flow rate and chamber design are the most important elements for a proper liquid-gas reaction	Bassani et al. (2016)

There have been inconsistent results with regard to glucose stability. A final methane content of 94.5% appeared in a batch study with glucose stability, while much lower methane contents were reported in other studies (Wahid et al., 2019). Such an issue could be overcome by moving the HM culture to a separate reactor to use *ex-situ* bio-upgrading. The lowest CH_4_ generation was shown when using grass as a substrate and a fish stone diffuser at pH 8 (Voelklein et al., 2019). Notably, the methane evolution rate was highest at 2.52 L L^−1^ d^−1^ with a ceramic diffuser and a grass substrate, compared to other types of diffusers used with the same type of reactor.

#### 2.2.2 Operational Considerations

Other issues with *in-situ* biological upgrading include the effects of H_2_ supply on microbial pathways and changes in reactor performance. Because the AD process requires anaerobic microorganisms for organics conversion to methane, the efficacy of the AD process essentially relies on the structure of the microbial communities in the reactor (Mulat et al., 2017; Martínez et al., 2019). Excessive H_2_ supply results in CO_2_ depletion, which increases the pH to 8.5 or higher. As such, it could negatively influence the operation of an AD reactor because strongly alkaline conditions are not suitable for methanogens, especially AMs (Luo et al., 2012; Bassani et al., 2015; Mulat et al., 2017; Wahid et al., 2019; Van et al., 2020).

Due to the complex nature of microbial composition in AD reactors, there is no single ideal microbial community used for an effective AD process and biological upgrading. In general, however, there are several dominating groups of microorganisms in an AD reactor, and their composition and ratio define the reaction pathways. HBM mainly relies on HMs, which belong to the orders *Methanobacteriales*, *Methanococcales*, *Methanomicrobiales*, and some representatives from *Methanosarcinales,* which are facultative hydrogenotrophs (Karakashev, et al., 2005; Sarmiento, et al., 2011). A more detailed taxonomic structure of HMs with some growth conditions (pH, temperature, G+C content) is summarized in Supplementary Table S1 in the Supplementary Information. Changes in dominating orders and their inner families, genera, and species ratio are caused by operational conditions, substrates, or other parameters (Luo and Angelidaki, 2012; Mulat et al., 2017; Martínez et al., 2019; Khan et al., 2022).

As a co-substrate, H_2_ itself has the potential to change microbial communities and influence the reactor performance, therefore, an optimum H_2_ supply must be achieved (Mulat et al., 2017). Thus, Luo and Angelidaki (2012) reported that the number of HMs increased with H_2_ addition, and the structure of the archaeal community altered. For example, in the reactor with H_2_ addition, a species *M. thermautotrophicus*, which can grow on H_2_, was detected. Interestingly, *M. thermautotrophicus* not only utilizes H_2_ and CO_2_ for methane production but also requires H_2_S for its growth (Strevett et al., 1995). Thus, this species can remove two contaminants from the biogas.

Wang et al. (2013) reported that H_2_ addition could lead to an increase of both homoacetogens and HMs. Thus, homoacetogenic bacterial genus *Treponema* and archaeal genus *Methanosaeta* dominated after H_2_ addition, together with HM genus *Methanoculleus*. In the study by Kim et al. (2013), AM genus *Methanosaeta* and HM genus *Methanospirillum* dominated the archaeal community after H_2_ supply.

Similar results were also reported in several studies. Martínez et al. (2019) expected HMs’ domination after H_2_ supply, but family *Methanosaetaceae* (AMs) remained the major methanogens in the reactor. However, after H_2_ supply, some HMs (such as genus *Methanospirillum*) increased in abundance. With additional H_2_ supply, the number of homoacetogens (such as bacterial families *Clostridiaceae* and *Eubacteriaceae*) has been shown to increase with an insignificant change in the ratio between AMs and HMs (Martínez et al., 2019).

Wu et al. (2021) reported that *Methanosaeta* (AMs) and *Methanobacterium* (HMs) were the two dominant archaeal genera. Among *Methanosaeta*, the major species was *M. harundinacea*; among *Methanobacterium*, the major species were *M. beijingense*, *M. petrolearium*, and *M. formicicum*. According to the study by Khan et al. (2022), even if hydrogenotrophic methanation was performed in the *in-situ* HBM, mostly by *Methanomicrobiales*, *Methanobacteriales*, and *Methanosarcinales* orders, the dominating species inside those groups varied according to the volume of supplied H_2_.

Thus, the HBM requires strict control of H_2_ supply to the system. When H_2_ supply is maintained properly, high efficiency is achieved in the conversion of CO_2_ to methane, and high system stability is performed. Thus, continuous monitoring of biogas composition is required, with installing an automatic gas analyzer and additional labor (Lecker et al., 2017).

### 2.3 Pros and Cons of *Ex-Situ* and Hybrid Biogas Upgrading Technologies

Both *in-situ* and *ex-situ* technologies have a few similar operational issues, such as H_2_ transfer and pH control. However, the *ex-situ* biogas upgrading is carried out in a separate system, where these issues could be resolved by applying a wider range of operational strategies for HMs. The most common way to improve the gas-liquid transfer of H_2_ (in almost 30 times), is the installation of a proper H_2_ gas diffuser (Bassani et al., 2017; Kougias et al., 2017; Voelklein et al., 2019; Ghofrani-Isfahani et al., 2021; Tang et al., 2021). Other strategies for improving the mass transfer rate are as follows. Burkhard et al. (2015) and Baransi-Karkaby et al. (2020) immobilized the microorganisms to avoid issues with gas-liquid transfer and to provide direct contact between the gas and microorganisms. Their studies resulted in 89–98% of CH_4_ content, respectively. Burkhard et al. (2015) found a correlation between increasing methane content by decreasing liquid recirculation rate. A recent study by Miehle et al. (2021) revealed that using a lab-scale bioreactor composed of 19 tubular dead-end membranes connected in series with a membrane pore size of 0.2 μM allowed a generation of gas with CH_4_ content of 99%. However, further study is needed to assess the potential for future applications.

Recent studies on *ex-situ* biological biogas upgrading are summarized in Table 2. In general, *ex-situ* processes showed higher efficiency and stability, rendering it a good option for new sites or sites with insufficient space for conventional systems construction. However, *ex-situ* reactor setups require a longer time for the adaptation and stabilization of microorganisms. While almost all studies were conducted on a small scale, no reviews are given on the maintenance of either the pure HM culture in an *ex-situ* reactor or the mixed culture of AMs and HMs in an *in-situ* reactor, meaning further investigation is needed.

**TABLE 2:** Comparison on the efficiency of *ex-situ* methanation systems.

Operation Conditions			Performance result					Comments	References
Reactor type	Diffuser type	Diffuser pore size, µM	pH	Temp., °C	CH_4_, %	CH_4_, L/L/d	Gas recirculation		
Up-flow (Batch)	Ceramic	—	8.5	55	92–96	—	No	The efficiency of CO_2_ conversion was related to gas recirculation	Voelklein et al. (2019)
Up-flow	Ceramic	—	7.1–8.2	55	15–85	—	Yes		
Up-flow in series, CSTR, Bubble column	Stainless steel	2	8	52 ± 1	79–98	—	yes, 12 L/h	CSTR showed the lowest final CH_4_ concentration	Kougias et al. (2017)
IBBR	—	—		37	89	—	Yes	The sludge was immobilized on a polymeric matrix; it prevents washing out of the biomass and allows recirculation ratios to increase	Baransi-Karkaby et al. (2020)
Up-flow	Al_2_O_3_, SiC	1.2; 0.5; 7; 14	6.95	55 ± 1	63–99	0.25–1.7	Yes	All diffusers showed a very high potential upgrading rate, but low stability	Ghofrani-Isfahani et al. (2021)
MBfR	19 tubular membranes	0.2	6–7	37	99	—	No	Mesophilic reactor with very high final CH_4_ concentration due to small pore size of the diffuser and series of columns	Miehle et al. (2021)
Up-flow	Stainless steel + alumina ceramic sponge; Al_2_O_3_ ceramic membrane	0.5; 2; 1.2; 0.4	∼7	55 ± 1	88–96	0.08–0.82	Yes	All membranes showed relatively similar upgrading efficiency, but different methane yield	Bassani et al. (2017)
Semi-continuous	—	—	5.5–9	55–70	28–75		No	Alkaline conditions were favorable for hydrogenotrophic methanogenesis, higher temperature (70°C)	Chen et al. (2021)
Semi-continuous	—	—	6, 7.5, 8.5	20–70	—	0.16–0.27	No	High temperature and alkali pH were the best conditions for *ex-situ* upgrading	Xu et al. (2020)
Batch	Quartz	Not specified	7, 8, 9	37	90.5	—	No	Investigation of different conditions showed that pH 8 and a short 5 min H_2_ injection time were the best for HM in the *ex-situ* upgrading reactor	Tang et al. (2021)
CSTR (Batch)	—	—	7.6	55	92–97		No	During the experiment, pH was not controlled and dropped to ∼6 and it affected the CH_4_ production. Proper pH control is required	Sekoai et al. (2020)
Trickle-bed	—	—	7.2–7.4	37 ± 0.5	96–98	1.2	No	Microorganisms are immobilized, thus contact with gas faze is supposed to be higher. It will provide better biogas upgrading	Burkhardt et al. (2015)

Hybrid system combines *in-situ* and *ex-situ* upgrading methods in one system. According to the study by Corbellini et al. (2018), a hybrid biogas upgrading system with both *in-situ* and *ex-situ* methods, along with thermophilic digestion was proposed. The system was composed of CSTR for the *in-situ* stage and UASB reactor for the *ex-situ* stage. The *in-situ* upgrading reactor was assisted with three stainless steel diffusers (2 µM pore size), while the reactor for the *ex-situ* process was assisted with a ceramic membrane. Hydrogen was directly injected into the first reactor providing *in-situ* upgrading, and the produced gas was subsequently moved for the *ex-situ* upgrading process. Although increasing CH_4_ content of up to 95% was found, with a pH maintained at 8.3–8.5, the accumulation of VFAs was confirmed in the *in-situ* process upon H_2_ injection, requiring the periodical control of VFAs. In contrast, a stable low level of VFAs was maintained in the *ex-situ* upgrading reactor with the high CH_4_ content. Improvement of H_2_ transfer efficiency was shown when using a ceramic membrane in the *ex-situ* process (Corbellini et al., 2018). When applying the hybrid process, high hydrogen utilization was achieved (up to 98%), indicating a possible application of the hybrid process to field plants.

### 2.4 Full-Scale Projects for Biological Biogas Upgrading

Conventional methods for biogas upgrading, which are mainly based on physical and chemical processes, are widely investigated and applied in practice. Multiple reviews on chemical/physical biogas upgrading projects and demonstration plants are available (Vartiainen, 2016; Ahmadi, 2017; Bailera et al., 2017; Thema et al., 2019). However, biological upgrading technology is still in the early stage of development and there is scarce information concerning large-scale field studies.

Although a number of studies have explored HBM, only a few pilot or full-scale applications have been tried. Some of the full-scale investigations are summarized. First, the MicrobEnergy Company conducted research on the *in-situ* biological biogas upgrading by adding H_2_ to an AD reactor in Schwandorf, Germany (Benjaminsson et al., 2013), increasing the methane content from 50 to 75%.

The BioCat project in Avedøre, Denmark, achieved 97% of methane content *via* biological upgrading technology in their demonstration unit of 4.8 m^3^ reactor volume (Power-to-Gas via Biological Catalysis, 2017). An *ex-situ* demonstration plant operated in Germany (IEA Bioenergy, 2018) showed 30 m^3^ of raw biogas per hour with 98% of methane content. In the study, a CSTR methanation tank with a working volume of 5 m^3^ was operating at a working temperature of 50–80°C and an internal pressure of 5–15 bar.

Jensen et al. (2018) investigated a special way of improving liquid-gas transfer. In their study, they applied a venturi-type injection system to a full-scale thermophilic digester, treating manure with *in-situ* HBM. The consumption rate of H_2_ in the study varied from 10 to 26%, indicating an incomplete reaction for CO_2_ conversion. This resulted in CH_4_ upgrading to 0.17–1.34% only, while potential upgrading could be 7%. Recirculation of the gas in the headspace of the reactor increased the consumption of H_2_. However, further investigation is necessary to evaluate the H_2_ injection method.

Store&Go project revealed three biogas upgrading operation sites. One of them, located in Solothurn, Switzerland, used HBM technology (Schlautmann et al., 2021). H_2_ was provided by proton exchange membrane electrolysis and CO_2_ was transported from a nearby wastewater treatment plant *via* pipeline. CO_2_ and H_2_ were converted to CH_4_ in a bubble-stirred column bioreactor with a temperature at 62°C and a pressure at 11 bar. The produced biomethane (more than 99% CH_4_ content) was injected into the urban gas distribution grid.

Despite its potential, further investigations of the biological upgrading technology are required, particularly for full-scale plants. Despite its long history and environmental and economic benefits, biogas/biomethane usage is still not widely considered as a sustainable energy source. Given the positive results of existing studies and the high technological potential of the HBM method, promoting biogas and biomethane production and usage is still encouraged.

## 3 HBM Upgrading as a Method to Promote Biogas Plants and Public Acceptance

Globally, there is growing awareness of greenhouse gas emissions, sustainable energy production, and the environmental impacts of fuel combustion among the public. However, it appears most countries do not consider biogas and biomethane as a sustainable energy source. The public acceptance of biogas varies among different countries and sometimes among neighborhoods, revealing different attitudes to biogas production and usage (Liu et al., 2013; Kemausuor et al., 2018; Xue et al., 2020).

The EU has led the use of upgraded biogas (IEA, 2018), and almost 500 conventional upgrading plants are operating in the EU as of 2019 (IEA, 2019; Schmid et al., 2019). There are conventional biogas upgrading plants in the United States, China, Canada, Brazil, South Korea, and Japan (Moon et al., 2019a; Moon et al., 2019b; IEA, 2019; Schmid et al., 2019; CIBiogás, 2021). On another hand, in areas with not developed or developing biogas markets, such as Eastern Europe, Balkan region, Central Asia, India, Australia, Latin America, Sub-Saharan Africa, the attitude to biogas usage usually varies from neutral to negative. More information about the status of biogas acceptance in selected countries and regions is summarized in Supplementary Table S2.

As shown from this maldistribution of the plants, still biogas plants are not widely applied, despite the apparent environmental and economic benefits of using biogas and upgraded biogas, which can be attributed to still lower public acceptance. Such lower public acceptance appears due to multiple reasons (e.g., improper standardization for biogas/biomethane quality, poor infrastructure, improper management), but the main limitation for biogas promotion is poor quality of produced biogas compared to natural gas (Pollmann et al., 2014; Garfí et al., 2016; Moreda, 2016; Kemausuor et al., 2018; Carlu et al., 2019; Mittal et al., 2019; Singh et al., 2019; Martinov et al., 2020; Abilmazhinov et al., 2021; Kuzhel et al., 2021; Zainutdinova et al., 2021). Thus, improved performance of biogas plants with higher methane gas output *via* applying HBM technology can significantly increase biogas plants promotion.

In areas with negative experience in biogas production in the past, HBM can be a good option to improve public and authorities’ attitude to biogas. For example, due to the energy crisis, there was the intensive implementation of AD reactors in the Latin America region in the 1970s (Garfí et al., 2016; Díaz-Vázquez et al., 2020). However, poor performance due to financial and technical issues, resulted in low support and most of those digesters stopped operating (Pérez et al., 2014; Garfí et al., 2016). Since Latin governments plan to promote small-size anaerobic digesters (Garfí et al., 2016), conventional upgrading systems are infeasible. Thus, by applying HBM upgrading it is possible to improve the performance of planned biogas plants, possibly increasing the public acceptance and supporting biogas plants.

In addition, HBM can be directly beneficial for the biogas market in developing green energy markets. Nagel and von Blottnitz (2021) stated that the construction and operation of large-scale (>1 MW) biomethane plants is more feasible in immature biogas markets due to higher energy values produced, followed by higher profits. In this case, due to lower capital investment, easier operation, and smaller size, the HBM becomes more attractive upgrading technology for large-scale sites compared to conventional systems.

Additionally, HBM biogas upgrading is considered a way to promote the developed biogas markets. For example, in Germany, where a significant amount of the biogas is produced from small- or medium-scale AD plants in rural areas, around 87% of upgrading facilities are performed on a large-scale (Daniel-Gromke et al., 2018). Even though farmers show positive attitude to biogas and biomethane plants (Emmann et al., 2013; Liu et al., 2013; Zemo et al., 2019), they tend to support only small and medium-scale plants (Zemo and Termansen, 2018; Zemo et al., 2019). Thus, due to its compactness HBM is considered the most optimal way to upgrade biogas on those sites (Daniel-Gromke et al., 2018; Liebetrau et al., 2021). Additionally, since HBM can be emphasized as a carbon-neutral process, additional economic and environmental benefits are foreseen, especially for regions with strict environmental policies.

**FIGURE 3:**
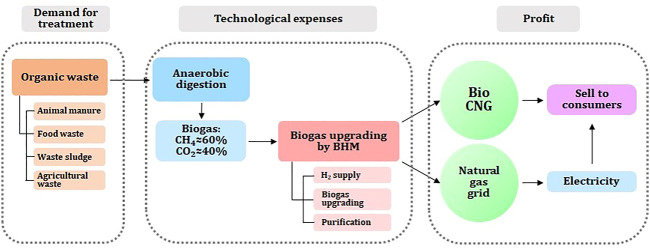
Business model for biogas utilization.

Moreover, by the development and application of HBM upgrading technology, it is possible to make biogas/biomethane the most sustainable green energy source. Since biogas is produced from organic wastes, and upgrading can replace natural gas, it is considered to be the perfect green and sustainable technology. Nonetheless, government policies together with public support are critical for the development of the biogas and biomethane plants (Kapoor et al., 2019; Nevzorova and Karakaya, 2020; Xue et al., 2020). A potential solution would be for governments and businesses to develop a business model for biogas utilization, which focuses on generating profits from gas and electricity and the sale of organic waste. It is possible to upgrade biogas to biomethane to inject in existing gas lines or to sell it as CNG for transportation and cooperation with city gas. As a proposed utilization business model, a fuel cell (Solid Oxide Fuel Cell, SOFC) profit model can be constructed for the supply of fuel for transportation by electricity and hydrogen reforming, as shown in Figure 3.

HBM biogas upgrading appears to be successfully applied in mature and immature biogas markets, in small, medium, and large-scale systems, in developed and developing regions. With the profits received from HBM technology application and the resultant support of the biogas and biomethane plants, it is possible to establish biomethane as a sustainable energy source.

## 4 Future Research Outlook

Biomethane is an attractive energy source when considering reducing climate change, developing zero-carbon policies and increasing economic profits from bioenergy. In most regions there is a positive attitude from locals towards biogas and biomethane usage. Nevertheless, several issues, including a lack of clear governmental policies and regulations; a limited number of technical experts; insufficient public education, and local construction issues, create hurdles for technology applications. More research is directed towards investigating biological biogas upgrading technologies, and positive propaganda of green energy such as biomethane is being used to convince residents and businesses of its benefits. Based on the literature, several future research directions have been identified, including 1) the effect of biofilm on CH_4_ generation in biogas upgrading systems; 2) performance evaluation of hybrid biogas upgrading; and 3) life cycle assessments of biogas upgrading.

Few studies have investigated if biofilm influences the performance of CH_4_ generation in biogas upgrading systems, especially the pathways and operating factors responsible for increasing CH_4_ production in biogas upgrading. One of the issues found in AD is low mass transfer efficiency (Matsumoto et al., 2012). To overcome the low mass transfer efficiency between the substrate and microorganisms, different types of fibrous biofilm carriers were tested in a study by Liu et al. (2017).

Among four types of fibrous biofilm carriers—polypropylene, polyester, polyamide, polyurethane fiber material—the polypropylene biofilm carrier system produced more biogas (∼45%) and methane (∼50%), compared to the control system (Liu et al., 2017). Similarly, polypropylene was seen to influence the start-up of methanogenic biofilm reactors, producing the highest biofilm concentrations with the highest removal of chemical oxygen demand and organic loading rate in anaerobic biofilm reactors (Habouzit et al., 2014). Despite ongoing research on biogas upgrading and the effects of biofilm on the AD performance, limited information has been shared about the effects of biofilm on CH_4_ generation in biogas upgrading.

While this review focused mainly on *in-situ* and *ex-situ* biological hydrogen methanation, especially recent updates on developments and prospects, a hybrid biogas upgrading system has not been studied enough and future research is needed to develop the technology, especially that which takes advantage of both *in-situ* and *ex-situ* biogas upgrading. While experimental data encourage further development of a hybrid system (95% CH_4_ content in the study of Corbellini et al., 2018), further pilot-scale studies are recommended to demonstrate applicability to the field. Analysis of the technology and its costs is also required as studies on a hybrid upgrade are limited to lab-based experimental or concept stages.

Lastly, there is limited information assessing *in-situ* vs. *ex-situ* biogas upgrading systems from case studies. Life cycle assessment (LCA) is a useful tool for assessing environmental impact. In a study by Starr et al. (2012), three biogas upgrading technologies (high-pressure water scrubbing (HPWS); alkaline with regeneration (AwR); and bottom ash upgrading (BABIU) were assessed using LCA. It was observed that, compared to water scrubbing, a higher impact on all LCA categories (global warming potential (GWP); eutrophication potential (EP); photochemical ozone creation potential (POCP) was found with AwR, whereas low GWP was found from AwR and BABIU through capturing and storing CO_2_ emissions (Starr et al., 2012). More studies on LCA as a tool for assessing biogas upgrading *via* HBM could assist in developing cost-effective and highly efficient biogas upgrading technologies for producing CH_4_.

## 5 Conclusion

Recently, the biogas conversion to a high-quality biomethane has been a strategic target in many countries. Although physical/chemical upgrading methods are at a high level of technological readiness, their wide application is limited. Biological upgrading *via* the HBM process is a new technology that creates new prospects for integrating different forms of renewable energy, including upgrading advances in energy storage and decoupling bioenergy production from biomass availability.

As far as the physical/chemical upgrade process is concerned, refining and upgrading processes in biogas production account for 60–70% of the total costs. As such, stabilizing the process for a long time and solving issues (e.g., methane concentration and efficacy of impurity removal) are essential. Further technological development is necessary to solve issues such as CH_4_ loss, environmental impact, maintenance costs, energy consumption in the separation process, CO_2_ separation conditions for solidification, and optimization to maintain appropriate partial pressure.

Recent research has been directed towards biogas upgrading using the HBM process. Upgrading *via* the HBM process is considered to be a low-cost, highly efficient way to upgrade biogas, and because CH_4_ content has the potential to reach up to 95% concentration through this process, it is possible to reduce CH_4_ purification costs by replacing existing technologies with biological biogas upgrading. The method, which uses HMs, consumes CO_2_ and H_2_ from the AD process and from outsourcing. As such, it consumes relatively little energy and has low costs.

However, an issue with an inefficient conversion rate of the gas to liquid during the H_2_ injection should be addressed. As long as the hydrogen economy is revitalized in the future and the H_2_ supply is stabilized through water electrolysis using renewable energy, the application of biogas upgrading *via* HBM process to field plants will be possible. With the HBM process, it is possible to create a sustainable energy source and promote biogas plants development. Despite the long history and high potential, in many regions the biogas market is undeveloped. Thus, the growing concern about renewable energy is a great opportunity to promote biogas production by biological biogas upgrading applications and to develop the green energy sector.

## Abbreviations

AMs, Acetoclastic Methanogens; AD, Anaerobic digestion; HBM, hydrogenotrophs-based biological methanation; COG, coke oven gas; CNG, compressed natural gas; CSTR, continuous stirred-tank reactor; EU, European Union; HFM, hollow fiber membrane; HMs, hydrogenotrophic methanogens; IBBR, immobilized biomethanation bioreactor; LCA, life cycle assessment; MBR, membrane bioreactor; MBfR, membrane biofilm reactor; Mtoe, million tonnes of oil equivalent; SAOB, syntrophic acetate-oxidizing bacteria; UASB, up-flow anaerobic sludge blanket; VFAs, volatile fatty acids.
