# Supplementary material for: Hydrogenotrophs-Based Biological Biogas Upgrading Technologies
Source: Front Bioeng Biotechnol. 2022 Apr 25;10:833482. doi: 10.3389/fbioe.2022.833482 (PMC9085624; doi:10.3389/fbioe.2022.833482)
Supplement: Supplementary file 1 [file DataSheet1.PDF]

Supplementary information

Table S1. HMs taxonomic structure

Order	Family	Genus	Species	Type strain	Operational range			References
					pH	Temp, ºC	G+C, mol%	
Methanobacteriales	Methanobacteriaceae	Methanobacterium	M. formicicum	MF (DSM 1535)	7-7.5	30-45	38-42	Garcia et al., 2006
			M. alcaliphilum	WeN4 (DSM 3387)	8-9	37	57	
			M. bryantii	M.o.H. (DSM 863)	6.5-7.5	30-45	33-38	
			M. congolense	C (DSM 7095)	7.2	37-42	39.5	
			M. espanolense	GP9 (NRC 5912 or DSM 5982)	5-6.5	30-45	34	
			M. ivanovii	Ivanov (DSM 2611)	7-7.5	30-45	37	
			M. oryzae	FPi (DSM 11106)	7	40	31	
			M. palustre	F (DSM 3108)	6.5-7.5	30-45	34	
			M. subterraneum	A8p (DSM 11074)	7.8-8.8	20-40	54.5	
			M. uliginosum	P2St (DSM 2956)	5-7.5	30-45	30-34	
		Methanobrevibacter	M. ruminantium	M1 (DSM 1093)	6.3-6.8	37-39	30.6	
			M. acididurans	ATM (DSM 15163)	6	35	nd	
			M. arboriphilus	DH1 (DSM 1125)	7.5-8	30-37	25.8	
			M. curvatus	RFM-2 (DSM 2462)	7.1-7.2	30	nd	
			M. cuticularis	RFM-2	7.7	37	nd	
			M. filiformis	RFM-3 (DSM 11501)	7-7.2	30	nd	
			M. gottschalkii	HO (DSM 11977)	7	37	29	
			M. oralis	ZR (DSM 7256)	6.9-7.4	35-38	28	
			M. smithii	PS (DSM 861)	6.9-7.4	nd	30-31	
			M. thaueri	CW (DSM 11995)	7	37	38	
			M. woesei	GS (DSM 11979)	7	37	31	
		Methanothermobacter	M. thermautotrophicus	ΔH (DSM 1053)	7.2-7.6	65-70	49	
			M. defluvii	ADZ (DSM 7466)	nd	nd	62.2	
			M. marburgensis	DSM 2133	nd	nd	47.6	
	Methanothermaceae	Methanothermus	M. fervidus	V24S (DSM 2088)	6.5	80-85	33	
			M. sociabilis	Kfl-F1 (DSM 3496)	6.5	88	33	

Abbreviation: nd, not determined

Table S1. HMs taxonomic structure (continued)

Order	Family	Genus	Species	Type strain	Operational range			References
					pH	Temp, ºC	G+C, mol%	
Methanomicrobiales	Methanomicrobiaceae	Methanomicrobium	M. mobile	DSM 1539 (ATCC 35094)	6.1-6.9	30-45	49	Bonin and Boone, 2006
		Methanolacinia	M. paynteri	G-2000 (DSM 2545 or ATCC 33997)	6.6-7.3	20-45	44	
		Methanogenium	M. cariaci	JR1 (DSM 1497, ATCC 35093 or OCM 155)	6-7.6	15-35	52	
			M. frigidum	Ace-2T (SMCC 459W or OCM 469)	6.3-8	0-17	52	
			M. frittonii	FR-4 (DSM 2832 or OCM 200)	6-8.25	26-62	49.2	
			M. organophilum	CV (DSM 3596 or OCM 72)	6.4-7.3	30-35	46.7	
		Methanoculleus	M. bourgense	MS2 (DSM 3045, ATCC 43281 or OCM 15)	5.5-8	30-50	59	
			M. marisnigri	JR1 (DSM 1498 or ATCC 35101)	5.7-7.6	15-45	61.2	
			M. oldenburgensis	CB-1 (DSM 6216)	6.5-8.5	25-50	47.6-49.6	
			M. olentangyi	RC/ER (DSM 2772, ATCC 35293 or OCM 52)	nd	30-45	54.4	
			M. palmolei	INSLUZ T (DSM 4273T)	6.5-8	22-50	59-59.5	
			M. thermophilicum	CR-1 (DSM 2373, ATCC 33837 or OCM 174)	6.7-7.2	55-60	56-60	
		Methanoplanus	M. endosymbiosus	DSM 3599	6.3-7.8	16-36	38.7	
			M. limicola	M3 (DSM 2279,ATCC 35062 or OCM 101)	6.5-7.5	17-41	47.5	
			M. petrolearius	SEBR 4847T (DSM 11571 or OCM 486)	5.3-8.4	25-45	50	
		Methanofollis	M. liminatans	GKZPZ (DSM 4140)	7	25-45	59.3-60.5	
			M. tationis	DSM 2702	6.3-8.8	25-45	54	
	Methanocorpusculaceae	Methanocorpusculum	M. bavaricum	SZSXXZ (DSM 4179 or OCM 127)	7	15-45	47.7-51	
			M. labreanum	ZT (DSM 4855, ATCC 43576, or OCM 1)	6.5-7.5	25-40	50	
			M. parvum	XII (DSM 3823, ATCC 43721 or OCM 63)	6.8-7.5	20-40	48.5	
			M. aggregans	Mst (DSM 3027 or OCM 21)	6.2-7.5	27-38	52	
			M. sinense	CHINAZ (DSM 4274 or OCM 128)	7	15-45	52	
	Methanospirillaceae	Methanospirillum	M. hungatei	JF1 (DSM 864, ATCC 27890 or OCM 16)	6.6-7.4	30-37	45-49	
	Genus incertae sedis	Methanocalculus	M. halotolerans	SEBR 4845 (OCM 470)	7-8.4	24-50	55	
			M. pumilus	MHT-1 (DSM 12632 or JCM 10627)	7	24-45	51.9	

Abbreviation: nd, not determined

Table S1. HMs taxonomic structure (continued)

Order	Family	Genus	Species	Type strain	Operational range			References
					pH	Temp, ºC	G+C, mol%	
Methanococcales	Methanococcaceae	Methanococcus	M. maripaludis	JJ (DSMZ 2067 or OCM 175)	6.5-8	20-45	33-35	Whitman and Jeanthon, 2006
			M. voltae	PS (ATCC 33273, DSMZ 1537 or OCM 70)	6.5-8	20-45	29-32	
			M. vannielii	SB (ATCC 35089, DSMZ 1224 or OCM 148)	6.5-8	20-45	32.5	
		Methanothermococcus	M. okinawensis	IH 1 (JCM11175 or DSM 14208)	4.5-8.5	40-70	nd	
			M. thermolithotrophicus	SN1 (DSM2095, ATCC35097, JCM10549 or OCM 138)	4.9-9.8	17-70	31-32	
	Methanocaldococcaceae	Methanocaldococcus	M. fervens	AG86 (DSM4213)	5.5-7.6	48-92	33	
			M. infernus	ME (DSM11812)	5.25-7	55-91	33	
			M. jannaschii	JAL-1 (DSM 2661 or ATCC 43067 or JCM 10045 or OCM 168)	5.2-7	50-91	31	
			M. vulcanius	M7 (DSM 12094 or ATCC700851)	5.25-7	49-89	31	
		Methanotorris	M. igneus	Kol5 (DSM5666)	5-7.5	45-91	31	
Methanosarcinales	Methanosarcinaceae	Methanosarcina	M. barkeri	MS (DSMZ 800 or OCM38)	6.5-7.5	25-50	39-44	Kendall and Boone, 2006
			M. mazeii	S6 (DSMZ 2053 or OCM 26)	5.8-8	25-45	42	
			M. vacuolata	Z-761 (ATCC 35090 or DSMZ 1232 or OCM 85)	6-8	20-45	36	
			M. lacustris	ZS (DSM 13486 or VKM B-2268)	4.5-8.5	1-35	43.4	

Abbreviation: nd, not determined

Table S2. Status of the biogas sector in selected countries and regions

Region	Country / Region	Attitude to biogas	Comments	Biogas facilities number		Energy production from biogas	References
				Biogas plants	Upgrading plants		
Europe	EU+UK	Positive/Negative	Positive attitude in countries with developed biogas sector (e.g. Germany, Denmark, UK, Sweden, France, Finland, and Estonia). Significant governmental support for green energy sector development. Significant public support in rural areas, relatively high acceptance in urban areas.<br>In some regions low trust of locals in green energy policies, ineffective biogas selling strategies lead to negative attitudes (e.g. Poland, Croatia, Romania)	~20000	580	61 TWh	Chasnyk, 2015;<br>IEA, 2019;<br>Scarlat et al., 2018;<br>Schmid et al., 2019;<br>Zemo et al., 2019
	Russia	Negative	Absence of supporting policies for bio-energy sector development due to relatively low prices for fossil fuels. Low level of concern about environment.	~100	-	n.d.	Boyprav et al., 2020;<br>Koshelev and Nurgaliev, 2016;<br>Zainutdinova et al., 2021
	Belarus	Neutral/Negative	Biogas is not considered a sustainable energy source. Biogas is produced from small- or middle-size AD and usually is incinerated.	29	-	38 MW	Krasouskaya and Karzhak, 2021
	Ukraine	Positive	Intention to develop independence in the energy sector, high potential for biomass and biogas. But government monopoly in energy sector creates difficulties in biogas plants development.	20	-	n.d.	Chasnyk et al., 2015;<br>Kuzhel et al., 2021
	Serbia	Neutral/Negative	Biogas is not considered an economically feasible and sustainable energy source. Due to low prices for natural gas, the biogas utilization is not considered feasible	8	-	15 MW	Martinov et al., 2020;<br>Pavlović et al., 2019
Asia	China	Positive	The government support for biogas sector development is high, same as public acceptance level, especially in rural areas.	over 100,000	73		Liu et al., 2013;<br>Schmidt et al., 2019
	Korea	Positive	The government policies are aimed to support and develop biomethane production and use, especially for transportation. The public acceptance level for biogas and biomethane usage as a transport fuel is high.	110	7	2798 GW	Jo et al., 2018;<br>Korean Ministry of Environment, 2020;<br>Moon et al., 2019
	India	Positive/Negative	Despite government attempts to promote the biogas sector, the public acceptance level remains low due to cultural, technical, social, and economic reasons	~5,000,000	-	n.d.	Mittal et al., 2018;<br>Mittal et al., 2019;<br>Singh et al., 2019
	Kazakhstan	Negative	Biogas is not considered a sustainable energy source. AD technology is not applied widely. Natural gas price is significantly lower than that of biogas.	5	-	n.d.	Abilmazhinov et al., 2021
	Uzbekistan	Positive	The government supports the AD sector development as biomass treatment technology coupled with bioenergy source production.	50	-	n.d.	Mamatkulova and Uzakov; 2018; 2021
Australia	Australia	Neutral/Negative	Biogas is not considered a sustainable energy source. Due to the absence of political will and poor economic policies, the biogas acceptance level remains low among citizens and authorities.	242	-	n.d.	Carlu et al., 2019;<br>Guerin et al., 2022;<br>Ngo et al., 2021
America	US	Positive	The government supports the biogas and biomethane plants development, but due to the lack of legislation and standardization there are issues with biogas market promotion.	~1500	77	n.d.	Schmidt et al., 2019
	Canada	Positive	The biogas sector in Canada is developed and biomethane production is considered a sustainable technology. Currently, around 16% of biogas is upgraded to biomethane and there projects to construct more upgrading plants.	279	11	196 MW	Canadian 2020 Biogas Market report, 2021;<br>IEA, 2019
	Latin America (except Brazil)	Neutral/Negative	Low acceptance of biogas, together with improper management led to biogas sector failure in the past (the 1970s), Currently, there are governmental activities to recover the biogas sector, but the attitude to biogas among citizens remains mostly negative.	n.d.	-	n.d.	Díaz-Vázquez et al., 2020;<br>Garfí et al., 2016
	Brazil	Neutral/Positive	The government set up goals for biogas sector development and successfully implemented them. Most of produced biomethane (73%) is used for power generation. The public support is unclear, but there is no definite negative attitude.	638	7	n.d.	CIBiogas, 2021;<br>Lima et al., 2018;<br>Dos Santos et al., 2018
Africa	Sub-Saharan Africa	Neutral/Negative	Despite high usage of traditional biomass and thus a high-expected level of biogas acceptance, poor infrastructure, economic issues, and lack of technical stuff lead to a low	n.d.	-	n.d.	Kemausuor et al., 2018

		acceptance level of biogas as an energy source among locals. However, governments are developing supporting policies for the biogas sector.

n.d. – no data

Soland et al. (2013) surveyed public biogas acceptance in Switzerland and found that the local attitude to biogas plants would be heavily influenced by trust in the government and economics such as benefits and costs (Fig. S1). Thus, providing information and educational materials to citizens and authorities is crucial to increasing support of biogas and biomethane. Emman et al. (2013) in their study investigated the effect of technical innovativeness on public acceptance and found,that personal innovativeness significantly affect the acceptance level.

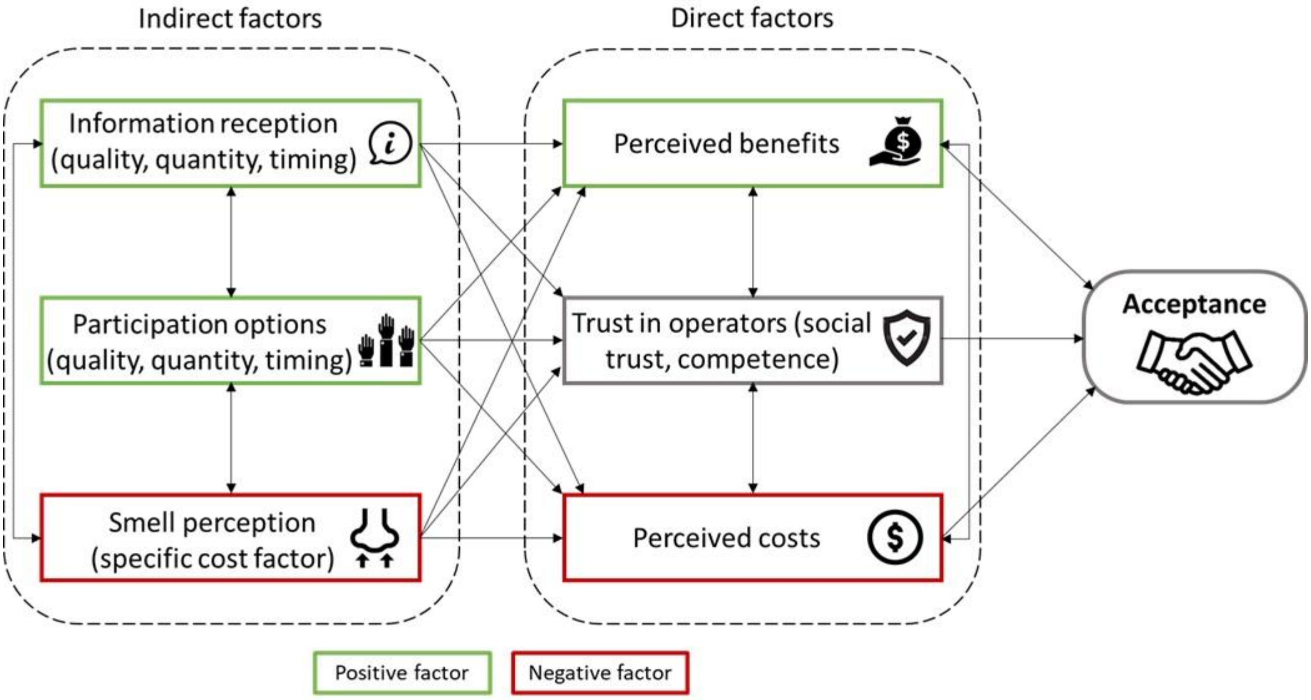

Figure S1. A hypothetical model of biogas plant acceptance (adapted from Soland et al., 2013)

References

Abilmazhinov, E. T., Shaiakhmetov, E. Y., Anibaev, S. N., Nurgaliyev, N. N., Shakerkhan, K. O., & Sailauov, D. M. (2021). Development of the biogas industry and prospects for the implementation of biogas plants in Kazakhstan. *Eurasian Physical Technical Journal*, 18 (3 (37)), 76-82. https://doi.org/10.31489/2021No3/76-82

Bonin, A. S., & Boone, D. R. (2006). The order methanobacteriales. The prokaryotes, 3, 231-243. doi: 10.1007/0-387-30743-5_11

Boyprav, L. V., Kalyamova, A. V., & Rumyantseva, A. V. (2020). Perspective of biogas use in Russia. In *The 14th International Scientific and Practical Conference "Environmental safety management system"*. – Yekaterinburg, 226-231. (In Russian) http://hdl.handle.net/10995/82624

Canadian 2020 Biogas Market Report SUMMARY. (2021) https://biogasassociation.ca/thankyou/2020_market_report_summary [Accessed on March 15 2022]

Carlu, E., Truong, T., & Kundevsk, M. (2019). Biogas opportunities for Australia. https://apo.org.au/node/243296 [Accessed on February 7, 2022]

Chasnyk, O., Sołowski, G., & Shkarupa, O. (2015). Historical, technical and economic aspects of biogas development: Case of Poland and Ukraine. *Renewable and Sustainable Energy Reviews*, 52, 227-239. https://doi.org/10.1016/j.rser.2015.07.122

Díaz-Vázquez, D., Alvarado-Cummings, S. C., Meza-Rodríguez, D., Senés-Guerrero, C., de Anda, J., & Gradilla-Hernández, M. S. (2020). Evaluation of biogas potential from livestock manures and multicriteria site selection for centralized anaerobic digester systems: The case of Jalisco, Mexico. *Sustainability*, 12(9), 3527. https://doi.org/10.3390/su12093527

Emmann, C. H., Arens, L., Theuvsen, L. (2013). Individual acceptance of the biogas

innovation: A structural equation model. *Energy Policy*, 62, 372-378. https://doi.org/10.1016/j.enpol.2013.07.083

Garfí, M., Martí-Herrero, J., Garwood, A., & Ferrer, I. (2016). Household anaerobic digesters for biogas production in Latin America: A review. *Renewable and sustainable energy reviews*, 60, 599-614. https://doi.org/10.1016/j.rser.2016.01.071

Garcia, J. L., Ollivier, B., & Whitman, W. B. (2006). The order methanomicrobiales. Prokaryotes, 3, 208-230. doi: 10.1007/0-387-30743-5_10

Guerin, T. F. (2022). Business model scaling can be used to activate and grow the biogas-to-grid market in Australia to decarbonise hard-to-abate industries: An application of entrepreneurial management. *Renewable and Sustainable Energy Reviews*, 158, 112090. https://doi.org/10.1016/j.rser.2022.112090

IEA Bioenergy Task 37. Upgrading Plant List 2019. 2019. http://task37.ieabioenergy.com/plant-list.html [Accessed on February 3, 2022]

Jo, J. H., Kim, W. (2018). Market potential of biomethane as alternative transportation fuel in South Korea. *Journal of Material Cycles and Waste Management*, 20(2), 864-876. https://doi.org/10.1007/s10163-017-0646-9

Kemausuor, F., Adaramola, M. S., Morken, J. (2018). A review of commercial biogas systems and lessons for Africa. *Energies,* 11(11), 2984. https://doi.org/10.3390/en11112984

Kendall, M., & Boone, D. (2006). The Order Methanosarcinales in Dworkin M., Falkow S., Rosenberg E., Schleifer K. H and Stackebrandt E. (eds) *The Prokaryotes*. doi: 10.1007/0-387-30743-5_12

Korean Ministry of Environment. Current status of biogasification facilities for organic waste resources in 2020. http://www.me.go.kr/home/web/policy_data/read.do?menuId=10265&seq=7683 [Accessed November 3, 2021] (In Korean)

Koshelev, V. M., & Nurgaliev, T. I. (2016). Risk assessment of biogas projects in Russia. *Izvestiya of Timiryazev Agricultural Academy*, (5) 108-118. (In Russian)

Krasouskaya, E., & Karzhak, A. (2021). Analysis of renewable energy sources using in Republic of Belarus.

Kuzhel, E. V., Talakh, L. A., & Shimchuk, O. P. (2021). Biogas - an unconventional energy source and more… In *The XV International Science Conference «Trends in the development of science and practice»*, December 27–29, Madrid, Spain. 436 p. (p. 403). (In Russian)

Lima, R. M., Santos, A. H., Pereira, C. R., Flauzino, B. K., Pereira, A. C. O., Nogueira, F. J., & Valverde, J. A. R. (2018). Spatially distributed potential of landfill biogas production and electric power generation in Brazil. *Waste Management*, 74, 323-334. https://doi.org/10.1016/j.wasman.2017.12.011

Mamatkulova, S., & Uzakov, G. (2018). Analysis of existing technologies and systems for biogas production. *TEXNOLOGIYALAR* № 4 (32) October-December, 27. (In Russian)

Mamatkulova, S., & Uzakov, G. (2021). Assessment of the Gross Potential of Local Waste Based on Geoinformation Systems for Bioenergy Production. *The Journal of CIEES*, 1(1), 34-39. https://doi.org/10.48149/jciees.2021.1.1.6

Martinov, M., Scarlat, N., Djatkov, D., Dallemand, J. F., Viskovic, M., & Zezelj, B. (2020). Assessing sustainable biogas potentials — case study for Serbia. *Biomass Conversion and Biorefinery*, 10(2), 367-381. https://doi.org/10.1007/s13399-019-00495-1

Mittal, S., Ahlgren, E. O., & Shukla, P. R. (2018). Barriers to biogas dissemination in India: A review. *Energy Policy*, 112, 361-370. https://doi.org/10.1016/j.enpol.2017.10.027

Mittal, S., Ahlgren, E. O., & Shukla, P. R. (2019). Future biogas resource potential in India: a bottom-up analysis. *Renewable Energy*, 141, 379-389. https://doi.org/10.1016/j.renene.2019.03.133

(a) Moon, H., Kwon, J., Park, H., Jeon, T., Shin, S., & Lee, D. (2019). A Study on Establishment of Technical Guideline of the Installation and Operation for the Biogas Utilization of Transportation and City Gas: Results of the Field Investigation. *Journal of the Korea Organic Resources Recycling Association*, 27(1), 77-85. (In Korean) https://doi.org/10.17137/korrae.2019.27.1.77

(b) Moon H., Lee, D., Kwon, J., Park, H., & Jeon, T. (2019). A study on the status of biogas upgrading facility and conversion effciency of biomethane in Korea. *Proceedings of the spring academic presentation of the Korean Society for Waste Recycling*, 108-108. (In Korean)

Ngo, T., Ball, A. S., & Shahsavari, E. (2021). The Current Status, Potential Benefits and Future Prospects of the Australian Biogas Sector. *Journal of Sustainable Bioenergy Systems*, 11(01), 14. doi: 10.4236/jsbs.2021.111002

Pavlović, T. M., Mirjanić, D. L., Radonjić, I. S., Stanković, A. M., & Piršl, D. S. 2019. Current state and perspectives of renewable energy sources use in Serbia.

dos Santos, I. F. S., Vieira, N. D. B., de Nóbrega, L. G. B., Barros, R. M., & Tiago Filho, G. L. (2018). Assessment of potential biogas production from multiple organic wastes in Brazil: Impact on energy generation, use, and emissions abatement. *Resources, Conservation and Recycling*, 131, 54-63. https://doi.org/10.1016/j.resconrec.2017.12.012

Scarlat, N., Dallemand, J. F., & Fahl, F. (2018). Biogas: Developments and perspectives in Europe. *Renewable energy*, 129, 457-472. https://doi.org/10.1016/j.renene.2018.03.006

Schmid, C., Horschig, T., Pfeiffer, A., Szarka, N., Thrän, D. (2019). Biogas upgrading: a review of National Biomethane Strategies and support policies in selected countries. *Energies*, 12(19), 3803. https://doi.org/10.3390/en12193803

Singh, S., Choudhary, B., Xavier, S., Roy, P., Bhagat, N., & Allen, T. (2019). Biogas Potential in India: Production, Policies, Problems, and Future Prospects. *Emerging Energy*

Alternatives for Sustainable Environment, 1-34. doi: 10.1201/9780429058271-1

Soland, M., Steimer, N., Walter, G. (2013). Local acceptance of existing biogas plants in Switzerland. *Energy Policy*, 61, 802-810. https://doi.org/10.1016/j.enpol.2013.06.111

Whitman, W. B., & Jeanthon, C. (2006). The sequencing of representative genes of the methane-producing archaea in the order Metha-nococcales suggests that this lineage is ancient and possesses a high degree of genetic diversity. For example, the mesophile Methanococcus. *Prokaryotes*, 3, 257-273. doi: 10.1007/0-387-30743-5_13

Zainutdinova, A. F., Sadykova, A. R., Ilgamova, L. F., & Mukhametova, I. V. (2021). Analysis of the prospects for the use of biogas in Russia. *International Journal of Humanities and Natural Sciences*, vol. 5-2 (56). (In Russian) doi: 10.24412/2500-1000-2021-5-2-181-183

Zemo, K. H., Panduro, T. E., Termansen, M. (2019). Impact of biogas plants on rural residential property values and implications for local acceptance. *Energy policy*, 129, 1121-1131. https://doi.org/10.1016/j.enpol.2019.03.008
